# Poster Session I - A141 IMPACT OF REFERRAL PATHWAY ON COLONOSCOPY OUTCOMES IN ADULTS AGED 75 YEARS AND OLDER

**DOI:** 10.1093/jcag/gwaf042.141

**Published:** 2026-02-13

**Authors:** V V Patel, V Kuttuva Sriganesh, A Tailor, M Rai

**Affiliations:** Gastroenterology, Queen’s University, Kingston, ON, Canada; Gastroenterology, Queen’s University, Kingston, ON, Canada; Gastroenterology, Queen’s University, Kingston, ON, Canada; Gastroenterology, Queen’s University, Kingston, ON, Canada

## Abstract

**Background:**

Colonoscopy remains a key assessment tool for several clinical situations. In adults aged 75 years and older, the risks and benefits of colonoscopy can be influenced by several factors, including comorbidities, frailty, and procedural risks associated with advanced age. Referral pathways vary between Direct-to-Endoscopy (DTE), where patients proceed directly to colonoscopy, and Clinic Assessment (CA), where pre-procedure evaluation allows individualized risk assessment and symptom review. While DTE may reduce delays, it may also bypass important optimization opportunities. There is limited evidence to guide which triage pathway should be used and it remains unclear if the type of pre-procedural assessment affects outcomes in patients above the age of 75.

**Aims:**

To determine whether differences in referral triage pathways affect colonoscopy quality indicators or safety outcomes in older adults

**Methods:**

A retrospective chart review included all colonoscopies performed at a single tertiary hospital from January 1, 2023, to December 31, 2024. Patients aged ≥75 years undergoing outpatient screening, diagnostic, or therapeutic colonoscopies were included. Emergent inpatient procedures were excluded. Data extracted from the electronic health record included demographics, referral pathway, indication, endoscopic outcomes, and adverse events. Categorical variables were analyzed using Chi-square or Fisher’s Exact tests.

**Results:**

During the assessment period, 859 patients were at least 75 years old at the time of colonoscopy. This represented 11.7% of all patients who had outpatient colonoscopies at this academic centre. From these cases, 503 patients (59%) were sent directly to endoscopy, while 356 patients (41%) were triaged to clinic assessment before their procedure. The indication for colonoscopy in the DTE cases was primarily for screening (71.6%), while the indication for CA patients was primarily diagnostic (64.3%). Both the DTE and CA groups had similar cecal intubation rates of 92.8% and 92.7% respectively (p = 0.93). Adverse events requiring hospitalization were rare in both the DTE and CA pathways (0.4% vs 0.3%, p = 1). Both triage pathways were also similar in terms of the percentage of patients who were reported to have poor or procedure-limiting bowel preparations (5.1% in DTE vs 6.8% in CA, p = 0.39).

**Conclusions:**

In adults aged 75 years and older, colonoscopy outcomes were comparable between DTE and CA triage referral pathways when patients were appropriately triaged. No differences were seen in cecal intubation rates, bowel preparation quality and adverse events. These findings suggest that with suitable patient selection, a direct-to-procedure model can be a safe and efficient option for some older adults without compromising procedural quality. We will be investigating further into clinically significant lesions found.

**Funding Agencies:**

None

